# Elucidating the effects of blue light and NaCl on flavonoid biosynthesis in detached *Lycium ruthenicum* leaves by UPLC-MS/MS and chemometrics

**DOI:** 10.1016/j.fochx.2026.103507

**Published:** 2026-01-10

**Authors:** Haitao Zeng, Wentao Yang, Hao Xu, Tiantian Chen, Guodong Wang, Tong Li, Zhubing Hu, Tao Zheng

**Affiliations:** a School of Biological Science and Engineering, Shaanxi University of Technology, Hanzhong 723001, Shaanxi, China; b College of Life Sciences, Engineering Research Center of High Value Utilization of Western China Fruit Resources of Ministry of Education, Shaanxi Normal University, Xi'an 710119, China; c School of Life Sciences, Henan University, Kaifeng 475001, Henan, China; d Qinba State Key Laboratory of Biological Resources and Ecological Environment (Incubation), Shaanxi Key Laboratory of Bio-resources, Collaborative Innovation Center for Comprehensive Development of Biological Resources in Qinba Mountain Area of Southern Shaanxi, Hanzhong 723001, Shaanxi, China

**Keywords:** *Lycium ruthenicum*, Blue light, NaCl osmotic stress, Flavonoids, UPLC-MS/MS

## Abstract

*Lycium ruthenicum* is rich in health-promoting anthocyanins and other flavonoids, however the effects of blue light (BL) and NaCl on their biosynthesis remained poorly understood. UPLC-MS/MS and chemometrics were utilized to investigate individual and combined effects of BL and NaCl treatments on flavonoids in detached L. *ruthenicum* leaves. BL significantly increased total anthocyanin content to 1.35 units·g^−1^ FW, 150 mM NaCl raised it to 2.64, and their combination further enhanced anthocyanin accumulation to 3.40. Comprehensive metabolomic profiling identified 495 flavonoids across different treatments. PCA and OPLS-DA revealed that BL and NaCl activated specific flavonoid biosynthesis pathways. BL preferentially enhanced quercetin derivatives and cyanidin 3-O-(6-p-coumaroyl)glucoside accumulation, whereas WL-NaCl markedly promoted the conversion of naringenin to eriodictyol, leading to dihydromyricetin biosynthesis. Combined treatments significantly stimulated the transformation of dihydrokaempferol into dihydromyricetin, and further boosted petunidins accumulation. These findings offered valuable strategies to enhance bioactive compounds production in L. *ruthenicum* for nutraceutical applications.

## Highlights

• BL and NaCl synergistically enhance anthocyanin accumulation in L. *ruthenicum* leaves.
• Metabolomics identified 495 flavonoids, indicating pathway-specific induction by treatments.
• BL promoted dihydrokaempferol conversion to dihydroquercetin, boosting quercetin production.
• NaCl facilitated naringenin conversion to eriodictyol, enhancing dihydromyricetin biosynthesis.
• BL and NaCl combined stimulated dihydrokaempferol transformation to dihydromyricetin.

## 1 Introduction

*Lycium ruthenicum* Murr., a medicinal and edible shrub belonging to the Solanaceae family, is primarily distributed in the arid and salinization regions of Northwest China (Du et al., 2024). This species has garnered considerable research interest owing to its notable nutritional and medicinal properties, which are largely attributed to a high abundance of bioactive compounds, particularly flavonoids and anthocyanins (Wang et al., 2018). The metabolic profile and medicinal value of L. *ruthenicum* are significantly influenced by environmental factors, with light exposure and soil salinity among the most critical (Chen et al., 2022). Consequently, ecological regulation strategies to enhance the content of its primary active components have become a key focus in the field.

Environmental factors, especially light quality and salt stress, are key elicitors of flavonoid accumulation in plants (Li et al., 2020; Liu et al., 2023). The regulation of environmental factors on plant secondary metabolism is a dynamic research field bridging food chemistry and plant physiology (Zheng et al., 2022). Light, a pivotal environmental factor, supplies energy and functions as a key developmental signal, regulating plant growth and developmental processes from germination to senescence (Li et al., 2022). Previous studies have demonstrated that blue light (BL; 450 nm, 5000 lx) significantly enhanced the total anthocyanin content in detached L. *ruthenicum* leaves compared with white light, reaching 1.88 units·g^−1^ FW (*P* < 0.05) (Zeng et al., 2023). Similarly, in pepper fruits, BL upregulated key anthocyanin biosynthetic genes, leading to enhanced anthocyanin accumulation (Liu et al., 2022). Consistent with these findings, BL also promoted anthocyanin biosynthesis in pear peel, accompanied by upregulation of both structural genes involved in the anthocyanin pathway and the key transcription factor *PpMYB10* (Tao et al., 2018). Flavonoids, frequently induced under osmotic stress, confer significant benefits to plant cells by acting as direct osmotic regulators and enhancing reactive oxygen species (ROS) scavenging capacity (Liu et al., 2025). Under sucrose-induced osmotic stress, the total anthocyanin content exhibited a biphasic response, characterized by an initial increase followed by a subsequent decline, peaking at 3.87 units·g^−1^ FW under 500 mM sucrose treatment (Zeng et al., 2023). In tobacco, the expression of genes associated with anthocyanin synthesis was markedly upregulated under salt stress, resulting in elevated anthocyanin production and directly contributing to ROS elimination and plant protection (Chen et al., 2019). Despite these insights, the specific effects of blue light in combination with NaCl stress on the flavonoid metabolism in L. *ruthenicum* leaves remain poorly understood.

Recent advances in analytical technologies, particularly UPLC-MS/MS approach, have greatly improved the comprehensiveness and accuracy of metabolomic profiling (Deng et al., 2025). Through UPLC-MS/MS analysis, detailed annotation of pigment metabolites revealed that red quinoa cultivars were enriched with kaempferol glycoside derivatives, whereas black cultivars predominantly accumulated quercetin derivatives (Qian et al., 2023). Integration of UPLC-Q-ToF-MS/MS with CIELAB colorimetry further identified 46 key pigment metabolites, including anthocyanins and flavonoids, in Davidson plum and native currant (Hay et al., 2025). Similarly, UPLC-MS/MS analysis of four wine grape cultivars enabled the identification of 43 anthocyanins and 66 non-anthocyanin flavonoids, highlighting marked differences in phenolic composition and color intensity (Bai et al., 2025). Although previous research had utilized UPLC-MS/MS to quantify specific anthocyanins in detached L. *ruthenicum* leaves under BL and sucrose-induced osmotic stress (Zeng et al., 2023), large-scale detection, identification, and quantification of flavonoids in L. *ruthenicum* leaves cultured under BL and NaCl treatments remained largely unexplored.

Therefore, a UPLC-MS/MS-based approach was utilized to systematically investigate the individual and synergistic effects of BL and NaCl on the composition and abundance of flavonoids in L. *ruthenicum* leaves. The aim was to elucidate the regulatory roles of BL and NaCl stress in flavonoid metabolism of L. *ruthenicum*, with special emphasis on how their synergistic action promoted flavonoid accumulation by modulating specific branches of the flavonoid biosynthetic pathway. The results provided a novel theoretical foundation and a practical technical strategy for designing efficient bioreactor systems to produce high-value flavonoid extracts from L. *ruthenicum*, thereby supporting the development of innovative functional foods.

## 2 Materials and methods

### 2.1 Materials and incubation condition

The L. *ruthenicum* seeds were harvested in mid-September 2024 from the Ningxia Academy of Agriculture and Forestry Sciences. The seeds were initially rinsed thoroughly with sterile water to remove surface-adhering impurities. Surface sterilization was performed by vortexing the seeds in 30 mL of 75% (*v*/v) ethanol for 30 s, followed by 3 washes with sterile water. Subsequently, the seeds were then soaked in 40 mL of 10% (v/v) sodium hypochlorite solution with continuous shaking for 15 min, after which they were rinsed 3 times with sterile water. Following sterilization, approximately 20 seeds were aseptically inoculated per culture bottle containing solid Murashige and Skoog (MS) medium using a sterile pipette tip. The medium was prepared by combining 4.74 g of MS powder, 25.00 g of sucrose, 7.00 g of agarose powder, and deionized water to 1 L. All culture bottles were incubated in an artificial climate-controlled growth chamber at 25 °C for 60 d under a 16/8 h light/dark photoperiod. Leaves harvested from the resulting sterile seedlings were subsequently applied to the following experiments.

### 2.2 NaCl and light quality treatments

#### 2.2.1 NaCl treatment

After 60 days of growth, leaves from the 4th to 5th node above the stem base were collected from well-developed, uniformly sized sterile seedlings. The leaves were then excised and cut into segments approximately 1 cm^2^ in size. Approximately 10 segments were placed onto the surface of solid MS medium per plate, which were supplemented with different concentrations of NaCl (50, 100, 150, 200, 250, and 300 mM). The leaves were cultured at 25 °C under a 16/8 h light/dark photoperiod with a light intensity of 4500 lx. Leaf tissues were harvested for analysis after 3, 5, 7, and 9 days of treatment.

#### 2.2.2 Different light quality treatments

Similarly, after 60 days of growth, leaves from the 4th to 5th node above the stem base from uniformly sized sterile seedlings were excised and segmented (∼1 cm^2^). Approximately 10 segments were placed onto the solid MS medium per plate. The plates were subsequently incubated under one of three light quality treatments, all maintained at 4500 lx and 50 W: white light (WL, standard growth chamber bulbs), BL (450 nm), or red light (RL, 650 nm). All cultures were maintained at 25 °C under a 16/8 h light/dark photoperiod for 7 days before subsequent analysis.

### 2.3 Determination of total anthocyanidin content

Leaf samples from different treatments were snap-frozen in liquid nitrogen and pulverized into powder. The powder was then transferred into a 2 mL centrifuge tube, and its weight was recorded. Subsequently, 300 μL of 1% (*v*/v) methanol-diluted hydrochloric acid was added to the tube, and the mixture was centrifuged at 3000 r/min for 3 min at 4 °C. To facilitate complete anthocyanin extraction, the mixture was shaken overnight at 100 r/min in the dark at 4 °C. After extraction, samples were centrifuged again at 12,000 r/min for 5 min under 4 °C. A 0.25 mL aliquot of the supernatant was combined with 250 μL of sterile water and 500 μL of chloroform into a 1.5-mL centrifuge tube, followed by centrifugation at 12,000 r/min for 5 min under 4 °C. Then, 350 μL of the resulting supernatant was mixed with 650 μL of 1% methanol-diluted hydrochloric acid solution into a new 1.50 mL centrifuge tube. Absorbance was measured at 530 nm and 657 nm using a 754-UV–Vis spectrophotometer (Shanghai Spectrum, Shanghai, China), and the average was taken. Measurements were performed in triplicate, and the average was calculated. Anthocyanin content per gram of fresh leaf weight was calculated according to the previously established (Yin et al., 2012): Total anthocyanin content (units·g^−1^ FW) = (A₅₃₀ - 0.25 × A₆₅₇) / fresh leaf weight (g).

### 2.4 Determination of flavonoids by UPLC-MS/MS

#### 2.4.1 Dry sample extraction

Leaf samples treated with different light conditions and NaCl concentrations were freeze-dried under vacuum in a lyophilizer (Scientz-100F) and subsequently ground into powder (MM400, Retsch) at 30 Hz for 1.50 min. An aliquot (50 mg) of powder was extracted with 1200 μL of a pre-cooled (−20 °C) 70% methanol aqueous solutions (chromatography-grade, Merck, Darmstadt, Germany) spiked with internal standards. Then, the mixture was vortex-mixed for 30 s, and this process was repeated every 30 min for 6 times. Subsequently, the mixture was centrifuged at 12,000 r/min for 3 min. The supernatant was carefully collected and filtered through a 0.22 μm microporous membrane (SCAA-104, ANPEL, Shanghai, China). The filtrate was transferred into an injection vial for subsequent UPLC-MS/MS analysis (Zhu et al., 2025).

#### 2.4.2 UPLC conditions

Flavonoid profile detection was conducted on an ExionLC™ AD UPLC system (AB SCIEX) equipped with a Agilent ‌ZORBAX SB-C18 column (1.8 μm, 2.1 mm × 100 mm). The mobile phase was composed of solvent A (ultra-pure water with 0.10% chromatography-grade formic acid, *v*/v) and solvent B (chromatography-grade acetonitrile containing 0.10% formic acid, v/v). The gradient elution program was applied as follows (Chu et al., 2020): starting with 95% A and 5% B; over 9.0 min, the ratio was linearly changed to 5% A and 95% B, and kept for 1.0 min; subsequently, the composition was returned to the initial conditions (95% A and 5% B) within 1.1 min and re-equilibrated for 2.9 min. The flow rate, column temperature, and injection volume were set at 0.35 mL/min, 40 °C, and 2.0 μL, respectively. The column effluent was directed into an ESI-QTRAP mass spectrometer for detection.

#### 2.4.3 ESI-Q TRAP-MS/MS

The ESI source was operated under the following conditions described previously (Chu et al., 2020). The source temperature was maintained at 500 °C, with ion spray voltages (IS) of +5500 V (positive mode) and − 4500 V (negative mode). Gas I, gas II, and curtain gas (CUR) pressures were set at 50.0 psi, 60.0 psi, and 25.0 psi respectively; and collision-activated dissociation (CAD) was maintained at a high setting. Nitrogen as the collision gas at a medium pressure were utilized into QQQ scans acquired as MRM experiments. For each MRM transition, the declustering potential (DP) and collision energy (CE) were individually optimized. Specific MRM transitions were monitored within designated time segments based on the elution profiles of the metabolites.

All flavonoid metabolite data were processed using Analyst 1.6.3 software (AB SCIEX, Canada). Flavonoids were identified by high-resolution MS analysis combined with a self-built MetWare Database, incorporating characteristic retention times and precursor ion *m*/*z* values. Chromatographic peaks were integrated and manually verified using MultiQuant software 3.02 (AB SCIEX, Canada). The relative content of each compound was expressed as its chromatographic peak area. The metabolite content data were processed using unit variance scaling (UV). The calculation followed the formula: Z = (X-μ)/σ, where μ denoted the mean and σ represented the standard deviation of the variable. OPLS-DA was performed using R (MetaboAnalystR 1.0.1) to enhance group separation. Differential metabolites were identified based on a combination of fold change with VIP values generated from the OPLS-DA model. Metabolites exhibiting a VIP value≥1 and a fold change (FC) ≥ 2 or ≤ 0.5 were screened as differential metabolites.

### 2.5 Data analysis

Significant differences across different treatments were assessed by one-way analysis of variance (ANOVA), followed by Tukey's post-hoc test for multi-comparisons, with statistical significance set at *p* < 0.05. All statistical analyses were carried out with SPSS 26 (IBM SPSS Inc., Chicago, IL, USA). Total anthocyanidin content data for all treatments were analyzed in triplicate and presented as mean ± standard error. Visualization plots, including multi-group histograms, categorical charts, segmented and combined heatmaps, volcano plots, scatter plots, and Venn diagrams, were generated from the Metware Cloud platform (https://cloud.metware.cn) (accessed on 20 October 2025).

## 3 Results

### 3.1 Effects of NaCl treatments on total anthocyanin content

**Fig. 1:**
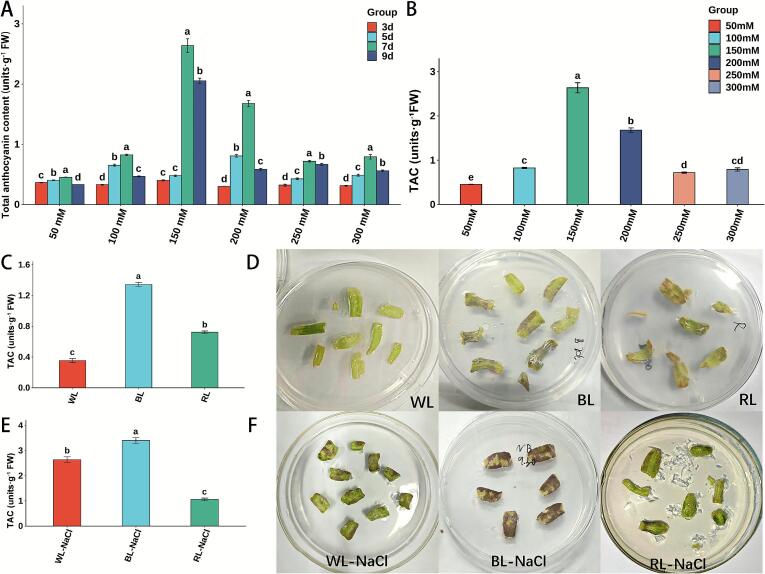
Effects of various treatments on anthocyanin accumulation in detached *L. ruthenicum* leaves. **A**: Anthocyanin content in leaves treated with different concentrations of NaCl over time. **B**: Anthocyanin levels under different NaCl concentrations on day 7 of treatment. **C**: Effects of light quality on anthocyanin content in detached leaves (WL, white light; BL, blue light; RL, red light). **D**: Visual color variations of leaves cultured under different light qualities. **E**: Anthocyanin content in leaves under combined treatment of light quality and 150 mM NaCl (WL-NaCl, White light and 150 mM NaCl; BL-NaCl, Blue light and 150 mM NaCl; RL-NaCl, Red light and 150 mM NaCl). **F**: Phenotypic coloration of leaves exposed to combined light quality and 150 mM NaCl. Error bars represented standard error from 3 biological replicates. Different lowercase letters in each plot represented the significant differences at the 0.05 level. (For interpretation of the references to color in this figure legend, the reader is referred to the web version of this article.)

Under 50 mM NaCl treatment, detached L. *ruthenicum* leaves developed purple pigmentation by day 7, with yellowing beginning from both ends by day 9. Anthocyanin content exhibited significant variation (*p* < 0.05) over the treatment period, displaying an initial increase followed by a decline, with a peak value of 0.45 units·g^−1^ FW on day 7 (Fig. 1A). At 100 mM NaCl, leaves turned purple coloration between days 5–7, and yellowing emerged at the cut ends by day 9. Anthocyanin levels also varied significantly (*p* < 0.05), reaching a maximum of 0.83 units·g^−1^ FW on day 7, which was 2.47-fold higher than that on day 3 (Fig. 1A). Under 150 mM NaCl treatment, leaves displayed strong purple pigmentation on day 7, appeared slightly yellowed and less turgid by day 9, and showed significant enlargement on days 7 and 9. From days 5 to 9, anthocyanin content varied significantly (*p* < 0.05), increasing initially and then decreasing, with the highest level observed on day 7 (2.64 units·g^−1^ FW), which was 6.5 times that on day 3 (Fig. 1A). With 200 mM NaCl, leaves turned purple on day 5, which intensified by day 7 accompanied by leaf curling; by day 9, curling worsened and partial chlorosis occurred. Anthocyanin content varied significantly (*p* < 0.05) throughout the treatment period (days 3–9), reaching a maximum on day 7 (1.68 units·g^−1^ FW), representing a 5.30-fold increase compared to day 3 (Fig. 1A).Under 250 mM NaCl, leaves displayed purple pigmentation and mild curling on day 5, which intensified by day 7 along with leaf thickening; by day 9, thickening became more pronounced, accompanied by partial chlorosis and browning at cut edges. Anthocyanin content peaked on day 7 (0.72 units·g^−1^ FW), following a significant rise-and-fall trend (*p* < 0.05) (Fig. 1A). Under 300 mM NaCl, leaves became purple and curled on day 5; by day 7, the purple coloration deepened, accompanied by thickening and cut-edge yellowing; by day 9, further thickening, yellowing, and whitening were observed, with some leaves senescing. Anthocyanin content reached its highest level on day 7 (0.79 units·g^−1^ FW), with significant differences (*p* < 0.05) during the treatment period (Fig. 1A).

In summary, NaCl treatments at various concentrations consistently promoted anthocyanin accumulation in detached L. *ruthenicum* leaves, with the highest content observed on day 7 across all treatment groups (Fig. 1A). A seven-day exposure to different NaCl concentrations significantly enhanced anthocyanin production, with the highest level (2.64 units·g^−1^ FW) occurring under 150 mM NaCl (Fig. 1B).

### 3.2 Effects of different light qualities treatments on total anthocyanin content

Significant differences in anthocyanin content were observed under different light qualities treatments (*p* < 0.05). The highest anthocyanin content was recorded under BL treatment, reaching 1.34 ± 0.03 units·g^−1^ FW, which was 3.79-fold and 1.86-fold higher than that under WL treatment (0.35 ± 0.03 units·g^−1^ FW) and RL treatment (0.72 ± 0.02 units·g^−1^ FW), respectively (Fig. 1C). Leaves under BL treatment exhibited a predominant purple pigmentation. In contrast, no notable color alteration was observed under WL treatment, while RL treatment led to a distinct darkening of leaf color. Additionally, both RL and WL induced yellowish areas at wound sites of leaves (Fig. 1D).

### 3.3 Effects of combined NaCl and different light qualities treatments on total anthocyanin content

After a 7-day of combined treatment with 150 mM NaCl under different light qualities, anthocyanin accumulation in detached leaves of L. *ruthenicum* was significantly affected (Fig. 1E). The highest anthocyanin content was observed under BL with 150 mM NaCl treatment (BL-NaCl), reaching 3.40 ± 0.11 units·g^−1^ FW. This value was 1.30-fold higher than that under WL with 150 mM NaCl (WL-NaCl; 2.64 ± 0.11 units·g^−1^ FW) and 3.21-fold higher than that under RL with 150 mM NaCl (RL-NaCl; 1.06 ± 0.05 units·g^−1^ FW). Leaves treated with BL-NaCl exhibited nearly complete purple pigmentation, whereas those under WL-NaCl treatment exhibited extensive purpling. In contrast, RL-NaCl treatment resulted in only slight darkening in restricted leaf areas (Fig. 1F).

Based on these findings, 4 treatments, WL, BL, WL-NaCl, and BL-NaCl, were selected for UPLC-MS/MS analysis to systematically investigate how BL and NaCl stress regulated anthocyanin accumulation in detached L. *ruthenicum* leaves.

### 3.4 Metabolomics component analysis

To further investigate the effects of BL and NaCl-induced osmotic stress, the composition and content of flavonoid metabolites were next assessed via UPLC/MS-MS in detached L. *ruthenicum* leaves treated with BL or WL and with 0 mM or 150 mM NaCl. Analysis of the quality control (QC) mixed samples in both positive and negative ion scanning modes exhibited that the ion current curves of metabolites were highly consistent across different batches, with matching retention times and peak intensities (**Fig. S1**). This indicated that the analytical system exhibited excellent mass spectrometry signal stability, and the obtained data were reliable with high reproducibility.

**Fig. 2:**
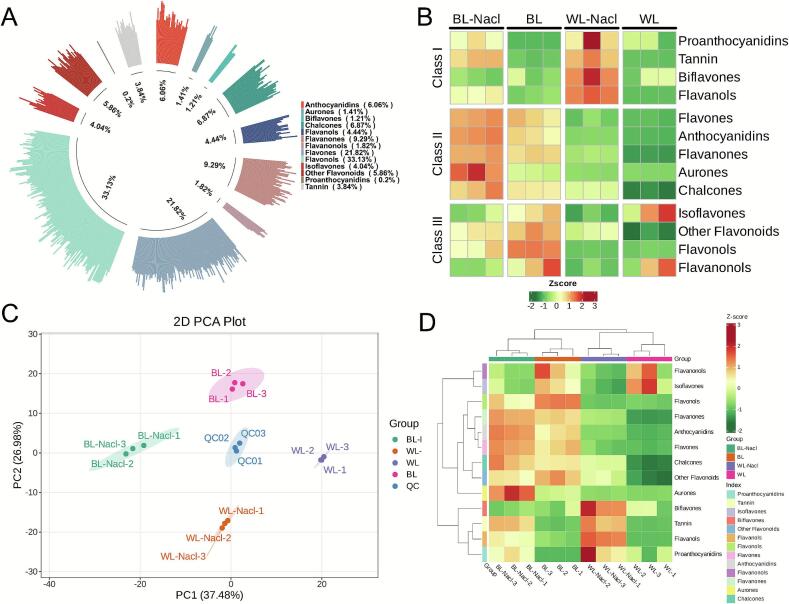
Comprehensive metabolomic characterization. **A**: Distribution of 495 identified metabolites across 13 distinct categories. **B**: Segmented heatmap of the relative abundance of the metabolites within each category. **C**: 2D-PCA Plot. **D**: Hierarchical clustering heatmap.

A total of 495 metabolites were detected across 4 treatment groups, which were classified into 13 categories (Table S1), 1 proanthocyanidin, 19 tannins, 20 isoflavones, 6 biflavonoids, 29 other flavonoids, 22 flavanols, 164 flavonols, 108 flavones, 30 anthocyanins, 9 dihydroflavonols, 46 dihydroflavones, 7 aurones, and 34 chalcones (Fig. 2A). Heatmap analysis revealed distinct accumulation patterns of flavonoid compounds under different treatments (Fig. 2B). Specifically, BL treatment increased higher levels of other flavonoids, flavonols, and dihydroflavonols. WL-NaCl treatment promoted the accumulation of proanthocyanidins, tannins, biflavonoids, and flavanols. The combined treatment of BL and NaCl stimulated the contents of flavones, anthocyanins, dihydroflavones, aurones, and chalcones. These results demonstrated that BL combined with NaCl treatment significantly enhanced the accumulation of anthocyanins in detached L. *ruthenicum* leaves.

### 3.5 Clustered behavior of detached L. ruthenicum leaves under different treatments

PCA results effectively revealed the overall distribution trends and intergroup distinctions among the different treatments. In the 2D-PCA score plot, the first two principal components, PC1 and PC2, accounted for 37.48% and 26.98% of the total variance, respectively (Fig. 2C), with a cumulative contribution rate of 64.46%, indicating that these components effectively captured the key characteristics distinguishing the samples under different treatments. The QC samples formed a tight cluster near the origin, confirming the stability of the analytical process and the high quality of the data. Clear separations were observed among the 4 treatment groups, which occupied distinct and non-overlapping regions in the score plot, reflecting substantial differences in their metabolite profiles. Furthermore, all biological replicates within the same treatment group clustered closely within the 95% confidence ellipses, demonstrating high reproducibility. Consistent with the PCA findings, the cluster heatmap analysis (Fig. 2D) further verified that the composition and levels of flavonoids in detached L. *ruthenicum* leaves varied considerably across the different treatment conditions.

### 3.6 Differential flavonoid accumulation

#### 3.6.1 Blue light-responsive flavonoids

Based on the OPLS-DA results, differential analysis of flavonoid metabolites in detached L. *ruthenicum* leaves under BL treatment was conducted. Differential metabolites were screened using thresholds of VIP > 1.0, and FC > 2.0 or < 0.5, to elucidate the regulatory effects of blue light on flavonoid accumulation.

**Fig. 3:**
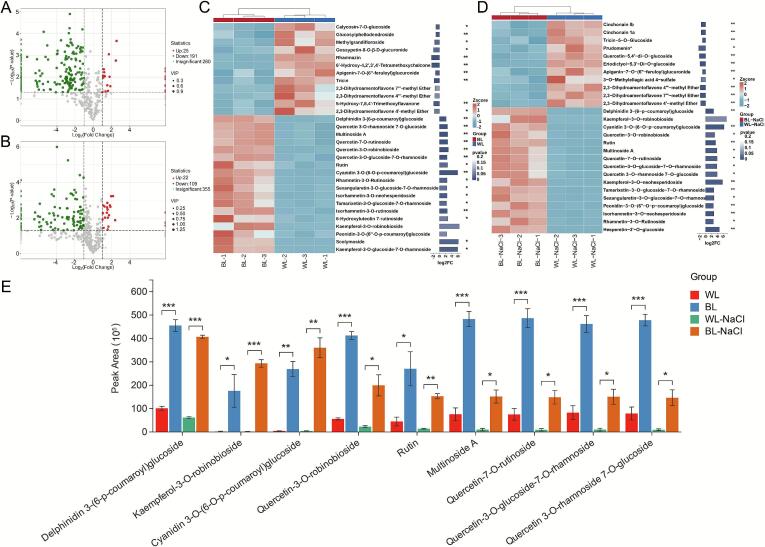
**A**: Volcano plot of differential metabolites under WL and BL treatments. **B**:Volcano plot of differential metabolites under BL-NaCl and WL-NaCl treatments. **C**: Combined heatmap of differential lipids exhibiting higher abundance in the WL_vs_BL comparison. **D**: Combined heatmap of differential lipids with increased abundance in the WL-NaCl_vs_BL-NaCl comparison. **E**: Bar chart showing significantly upregulated differential metabolites under blue light treatment based on intergroup testing (*, *p* < 0.05; **, *p* < 0.01; ***, *p* < 0.001). (For interpretation of the references to color in this figure legend, the reader is referred to the web version of this article.)

In the comparison between WL and BL treatments, 259 differential metabolites were identified (**Table S2**), of which 30 metabolites were up-regulated and 229 metabolites were down-regulated (Fig. 3A). In the BL-NaCl vs. WL-NaCl comparison, 187 differential metabolites were detected (**Table S3**), including 28 up-regulated and 159 down-regulated (Fig. 3B). Compared with WL treatment, BL treatment significantly suppressed the accumulation of calycosin-7-O-glucoside, glucosylphellodendroside, and methylgrandifloroside. Their contents under WL treatment were 2.17-, 2.22-, and 2.95-fold higher than those under BL treatment, respectively (Fig. 3C). Compared to WL-NaCl treatment, BL-NaCl treatment notably inhibited the accumulation of cinchonain Ib, cinchonain Ia, tricin-5-O-glucoside, and prudomenin (Fig. 3D). The contents of these metabolites in the WL-NaCl group were 2.90-, 2.84-, 2.14-, and 5.22-fold higher, respectively, than those in the BL-NaCl group. Furthermore, regardless of NaCl application, BL treatment consistently and significantly suppressed the accumulation of apigenin-7-O-(6″-feruloyl)glucuronide and 3 biflavones (2,3-dihydroamentoflavone 7″′-methyl ether, 2,3-dihydroamentoflavone 4″′-methyl ether, and 2,3-dihydroamentoflavone 4′-methyl ether).

Under BL treatment alone, significant increases were observed in the levels of isorhamnetin-3-O-rutinoside, scolymoside, and kaempferol-3-O-glucoside-7-O-rhamnoside (Fig. 3C). When combined with 150 mM NaCl treatment, the application of BL further promoted the accumulation of kaempferol-3-O-neohesperidoside and hesperetin-3’-O-glucoside (Fig. 3D). In BL-treated leaves, irrespective of NaCl conditions, pronounced enhancement was detected for several flavonols and anthocyanins, including delphinidin 3-(6-p-coumaroyl)glucoside, quercetin-7-O-rutinoside, multinoside A, quercetin-3-O-glucoside-7-O-rhamnoside, quercetin 3-O-rhamnoside 7-O-glucoside, quercetin-3-O-robinobioside, rutin, and cyanidin 3-O-(6-p-coumaroyl)glucoside (Fig. 3E). Notably, the delphinidin 3-(6-p-coumaroyl)glucoside exhibited a marked increase under both BL and BL-NaCl treatments, with levels rising by 4.50-fold and 6.60-fold compared to the WL and WL-NaCl treatments, respectively. Similarly, cyanidin 3-O-(6-p-coumaroyl)glucoside content exhibited a more substantial elevation in the BL and BL-NaCl groups, reaching values 60.59- and 77.81-fold higher than those in the corresponding WL and WL-NaCl controls.

Thus, BL treatment elicited a significant increase in the synthesis of flavonols and flavones. The flavone and flavonol biosynthesis pathway were notably up-regulated (**Fig. S2**), with pronounced accumulation of quercetin derivatives under BL treatment. The accumulation of cyanidin 3-O-(6-p-coumaroyl)glucoside was also further induced by BL treatment.

#### 3.6.2 NaCl stress-induced flavonoids

**Fig. 4:**
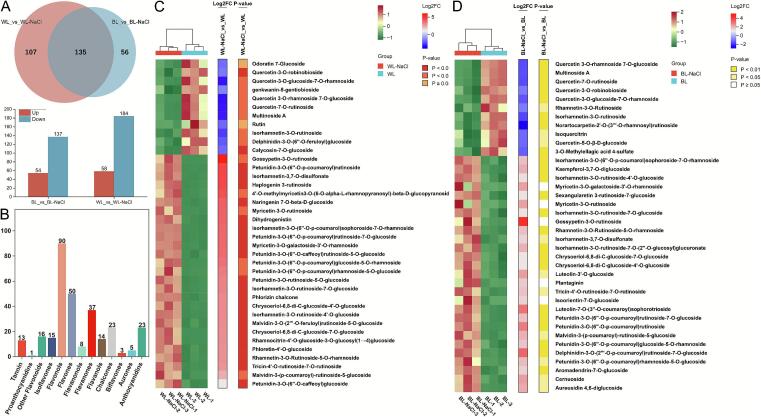
**A**: Venn diagram of differential metabolites and bar plots showing the numbers of differential metabolites between WL_vs_WL-NaCl and BL_vs_BL-NaCl comparisons. **B**: Classification of differential metabolites. **C**: Combined heatmap of differential lipids with higher abundance in the WL_vs_WL-NaCl comparison. **D**: Combined heatmap of differential lipids with higher abundance in the BL_vs_BL-NaCl comparison.

Under NaCl treatments, 298 differential metabolites were screened through comparative analysis of WL_vs_WL-NaCl and BL_vs_BL-NaCl (Fig. 4A). In the BL_vs_BL-NaCl comparison, 54 metabolites were up-regulated and 137 metabolites were down-regulated (**Table S4**), while the WL_vs_WL-NaCl comparison displayed 58 up-regulated and 184 down-regulated metabolites (**Table S5**). These differential metabolites were predominantly enriched in flavonoid compounds, including flavonols (90), flavones (50), flavanones (37), chalcones (23), and anthocyanins (23) (Fig. 4B). These compounds were associated with key biosynthetic pathways, comprising flavonoid biosynthesis, flavone and flavonol biosynthesis, and anthocyanin biosynthesis (**Fig. S3**).

In the WL_vs_WL-NaCl comparison, WL-NaCl treatment significantly suppressed the accumulation of several metabolites, including odoratin 7-glucoside, genkwanin-5-gentiobioside, rutin, delphinidin-3-O-(6″-O-feruloyl)glucoside, and calycosin-7-O-glucoside (Fig. 4C). Their contents under WL treatment were 2.87-, 2.09-, 3.13-, 3.91-, and 3.02-fold higher than those under WL-NaCl treatment, respectively. Similarly, under BL_vs_BL-NaCl conditions, NaCl application led to a marked reduction in the levels of rhamnetin-3-O-rutinoside, norartocarpetin-2′-O-(3‴-O-rhamnosyl)rutinoside, isoquercitrin, quercetin-5-O-β-D-glucoside, and 3-*O*-methylellagic acid 4-sulfate (Fig. 4D). The contents of these compounds under BL treatment were 2.04-, 6.09-, 2.50-, 2.54-, and 2.81-fold higher than those under BL-NaCl treatment, respectively. Furthermore, regardless of BL conditions, NaCl treatment consistently and significantly inhibited the accumulation of quercetin-7-O-rutinoside, quercetin 3-O-rhamnoside 7-O-glucoside, quercetin-3-O-glucoside-7-O-rhamnoside, multinoside A, and quercetin-3-O-robinobioside.

Compared to the WL treatment, the WL-NaCl treatment significantly promoted the accumulation of 3 anthocyanins, namely petunidin-3-O-(6”-O-caffeoyl)glucoside, petunidin-3-O-rutinoside-5-O-glucoside, and malvidin-3-O-(2″′-O-feruloyl)rutinoside-5-O-glucoside. Simultaneously, WL-NaCl treatments also markedly increased the contents of phlorizin chalcone, eriodictyol-7-O-(6″-acetyl)glucoside, eriodictyol-7-O-(6″-malonyl)glucoside 4’-*O*-methylmyricetin 3-O-(6-O-α-L-rhamnopyranosyl)-β-D-glucopyranoside, rhamnocitrin-4’-O-glucoside-3-O-glucosyl(1 → 4)glucoside, haplogenin 3-rutinoside, and naringenin 7-O-β-D-glucoside. Under BL treatment, the addition of NaCl substantially enhanced the accumulation of several flavonoids, including eriodictyol-7-O-(6″-acetyl)glucoside, eriodictyol-7-O-(6″-malonyl)glucoside kaempferol-3,7-O-diglucoside, luteolin-3’-O-glucoside, cernuoside, aureusidin 4,6-diglucoside, plantaginin, and aromadendrin-7-O-glucoside.

Moreover, NaCl treatment broadly enhanced the biosynthesis of numerous flavonoid constituents, independent of BL conditions. Specifically, the accumulation of the following compounds was significantly upregulated, comprising isorhamnetin-3-O-(6”-O-p-coumaroyl)sop-horoside-7-O-rhamnoside, chrysoeriol-6,8-di-C-glucoside-4’-O-glucoside, chrysoeriol-6,8-di-C-glucoside-7-O-glucoside, petunidin-3-O-(6”-O-p-coumaroyl)rutinoside, petunidin-3-O-(6”-O-p-c-oumaroyl)rhamnoside-5-O-glucoside, petunidin-3-O-(6”-O-p-cou-maroyl)rutinoside-7-O-glucosi-de, and malvidin-3-(p-coumaroyl)-rutinoside-5-glucoside.

In summary, NaCl treatment obviously inhibited the accumulation of quercetin derivatives, but markedly promoted that of petunidin, malvidin, rhamnetin, isorhamnetin, and glycosidic myricetin derivatives.

#### 3.6.3 Synergistic effect of blue light and NaCl treatments

The most remarkable findings of this study were the synergistic effects of the combined BL and NaCl treatments on flavonoids accumulation in detached L. *ruthenicum* leaves.

**Fig. 5:**
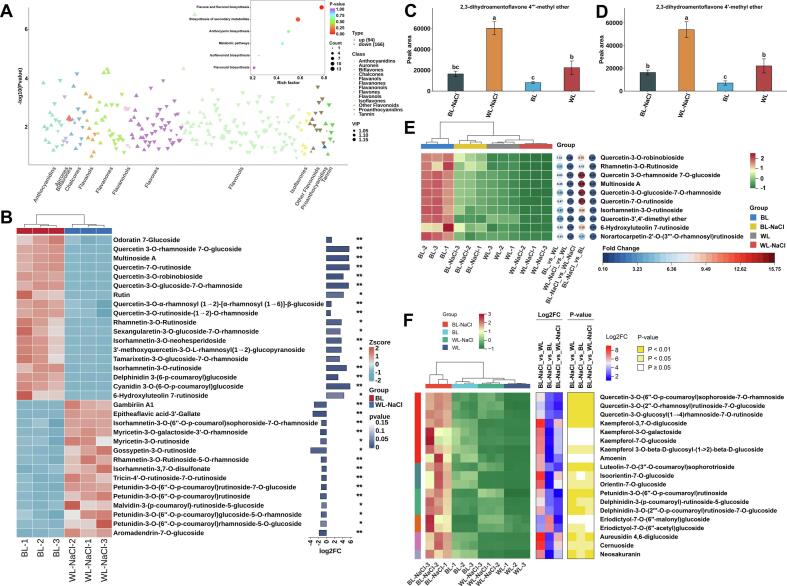
**A**: Categorical statistical scatter plot of differential compounds in the WL-NaCl _vs_BL comparison. **B**: Differential flavonoids with higher content in leaves under WL-NaCl and BL treatment. **C-D**: Comparison of the contents of 2,3-dihydroamentoflavone 4″′-methyl ether and 2,3-dihydroamentoflavone 4′-methyl ether. **E**: The different flavonoids with higher content in leaves under WL-NaCl, BL, WL, and BL-NaCl treatment. **F**: The combined heatmap of the 19 common differential metabolites under WL-NaCl, BL, WL, and BL-NaCl treatment.

In the WL-NaCl_vs_BL comparison, 94 up-regulated and 166 down-regulated differential metabolites were screened (**Table S6**). These metabolites were predominantly flavonols, flavones, and anthocyanidins, which were significantly enriched in the flavone and flavonol biosynthesis pathway, along with the anthocyanin biosynthesis pathway (Fig. 5A).

Compared to the BL treatment, WL-NaCl treatment significantly enhanced the accumulation of gambiriin A1, epitheaflavic acid-3’-Gallate, isorhamnetin-3-O-(6”-O-p-coumarol)sophoroside-7 -O-rhamnoside, 4’-*O*-methylmyricetin3-O-(6-O-alpha-L-rhamnopyranosyl)-beta-D-glucopyranoside, myricetin-3-O-galactoside-3’-O-rhamnoside, myricetin-3-O-rutinoside, gossypetin-3-O-rutinoside, rhamnetin-3-O-Rutinoside-5-O-rhamnoside, isorhamnetin-3,7-O-disulfonate, tricin-4’-O-rutinoside-7-O-rutinoside, petunidin-3-O-(6”-O-p-coumaroyl)rutinoside, petunidin-3-O-(6”-O-p-coumaroyl)rutinoside-7-O-glucoside, petunidin-3-O-(6”-O-p-coumaroyl)glucoside-5-O-rhamnoside, petunidin-3-O-(6”-O-p-coumaroyl)rhamnoside-5-O-glucoside, malvidin-3-(p-coumaroyl)-ru‑tinoside-5-glucoside, aromadendrin-7-O-glucoside (Fig. 5B).

In contrast, BL treatment promoted the accumulation of odoratin 7-Glucoside, quercetin 3-O-rhamnoside 7-O-glucoside, multinoside A, quercetin-7-O-rutinoside, quercetin-3-O-robinobioside, quercetin-3-O-glucoside-7-O-rhamnoside, rutin, quercetin-3-O-α-rhamnosyl(1 → 2)-[α-rhamnosyl (1 → 6)]-β-glucoside, quercetin-3-O-rutinoside-(1 → 2)-O-rhamnoside rhamnetin-3-O-ruti-noside, sexangularetin-3-O-glucoside-7-O-rhamnoside, isorhamnetin-3-O-neohesperidoside, 3′-methoxyquercetin-3-O-L-rhamnosyl(1 → 2)-glucopyranoside, tamarixetin-3-O-glucoside-7-O-r-hamnoside, isorhamnetin-3-O-rutinoside, delphinidin 3-(6-p-coumaroyl)glucoside, cyanidin 3-O-(6-O-p-coumaroyl)glucoside (Fig. 5B).

In comparison with WL treatment, WL-NaCl treatment significantly increased the accumulation of biflavones, namely 2,3-dihydroamentoflavone 4″′-methyl ether (Fig. 5C), and 2,3-dihydroamentoflavone 4′-methyl ether (Fig. 5D), reaching levels 2.69- and 2.44-fold higher, respectively. Conversely, BL treatment markedly reduced the contents of these 2 flavonoids. And, their levels under WL-NaCl treatment were 7.42- and 7.61-fold higher than those under BL treatment. Under the combined BL and NaCl treatment, their levels were significantly lower than those under WL-NaCl treatment, but still considerably higher than those under the BL treatment. These findings indicated that WL-NaCl treatment notably promoted the accumulation of 2,3-dihydroamentoflavone 4″′-methyl ether, and 2,3-dihydroamentoflavone 4′-methyl ether.

Compared to WL treatment, BL treatment significantly promoted the accumulation of 8 flavonols metabolites [quercetin-3-O-robinobioside, quercetin-3-O-rhamnoside-7-O-glucoside, quercetin-3-O-(4”-O-glucosyl) rhamnoside, quercetin-3-O-glucoside-7-O-rhamnoside, quercetin-7-O-rutinoside, rhamnetin-3-O-rutinoside, isorhamnetin-3-O-rutinoside, quercetin-3′,4′-dimethyl ether] and 2 flavones [6-hydroxyluteolin-7-rutinoside, normangiferin-2’-O-(3″′-O-rhamnosyl) rutinoside]. In contrast, WL-NaCl treatments led to a marked decrease in the contents of these compounds. Under combined NaCl and BL treatment, the levels of these 8 flavonols remained significantly lower than those under BL treatment, yet still substantially higher than those under WL-NaCl treatment. These results indicated that BL treatments effectively enhanced the accumulation of quercetin derivatives, isorhamnetin-3-O-rutinoside, and 6-hydroxyluteolin-7-rutinoside (Fig. 5E).

The combined treatment with BL and NaCl significantly enhanced the accumulation of 8 flavonols, 3 flavones, 3 anthocyanins, 2 flavanones, 2 aurones, and 1 chalcone (**Table S7**). Compared to the WL-NaCl treatments, the BL-NaCl treatment significantly increased the contents of kaempferol-3,7-O-diglucoside, kaempferol-3-O-galactoside, kaempferol-7-O-glucoside, and kaempferol 3-O-beta-D-glucosyl-(1- > 2)-beta-D-glucoside, with increases exceeding 17.00-fold (Fig. 5F). Relative to BL treatment, the increases in these components were approximately 3.00-fold. Meanwhile, the contents of isoorientin-7-O-glucoside and orientin-7-O-glucoside under the BL-NaCl treatment were 2.60- and 2.65-fold of those under BL treatment, and 49.48- and 31.02-fold of those under WL-NaCl treatment, respectively. The accumulation of petunidin-3-O-(6″-O-p-coumaroyl)rutinoside was markedly enhanced under the BL-NaCl treatment compared with either treatment alone, showing increases to 2.31- and 6.58-fold of the levels observed under BL and WL-NaCl treatments, respectively. Under WL-NaCl treatment, the contents of eriodictyol-7-O-(6″-malonyl)glucoside and eriodictyol-7-O-(6″-acetyl)glucoside increased to 3.46- and 2.43-fold, respectively, compared with those under WL treatment. In contrast, BL treatment reduced their contents to only 0.10- and 0.12-fold of the WL treatment. However, BL-NaCl treatment markedly promoted the accumulation of these two aurones, reaching 2.94- and 4.88-fold of the WL-NaCl treatment, and 106.98- and 96.51-fold of the BL treatment, respectively. Additionally, the content of neosakuranin following BL-NaCl treatment was 18.78- and 2.30-fold that under WL-NaCl and BL treatments, respectively. The combined treatment also markedly enhanced the accumulation of kaempferol-3,7-O-diglucoside, with its content reaching 3.74- and 54.82-fold of the contents observed under BL and WL-NaCl treatments, respectively.

## 4 Discussion

### 4.1 Effects of light quality and osmotic stress on anthocyanin accumulation

High concentrations of NaCl could induce osmotic stress and ion toxicity in plants, leading to the accumulation of reactive oxygen species (ROS) and consequent oxidative damage (van Zelm et al., 2020). Anthocyanins function as osmotic regulators in plant responses to osmotic stress and effectively scavenge excess ROS due to their antioxidant capacity (Lu et al., 2025). Numerous studies have demonstrated that anthocyanin content in plant cells increased significantly under osmotic stress treatments (Pei et al., 2025). For instance, anthocyanin content in tomato hypocotyls and cotyledons increased by 51% and 73%, respectively, after 7 days of treatment with 100 mM NaCl compared to the control (Eryılmaz, 2006). Similarly, flavonoid biosynthesis and anthocyanin content were significantly enriched in fruit of L. *ruthenicum* treated with 300 mM NaCl (Wang et al., 2023). Our previous work demonstrated that detached L. *ruthenicum* leaves treated with 500 mM sucrose exhibited a significantly increase in total anthocyanins content (Zeng et al., 2023). This study further revealed that anthocyanin content increased significantly within the 50–150 mM NaCl range. However, beyond 150 mM NaCl, anthocyanin content declined markedly, due to severe disruption of cellular structure and interference with normal metabolic functions caused by intense NaCl stress. The peaked anthocyanin content in detached L. *ruthenicum* leaves was observed at 150 mM NaCl treated with 7 days. This finding was consistent with previous research, indicating that L. *ruthenicum* leaves enhanced anthocyanin biosynthesis to improve stress resistance under osmotic stress.

Light (including intensity, quality, and photoperiod) serves as a key signaling factor modulating multiple stages of plant growth and development (Chang et al., 2025). Different light qualities exert distinct regulatory effects on anthocyanin synthesis (Zhou et al., 2022). Previous experimental results indicated that BL treatment induced the highest anthocyanin content in detached *L. ruthenicum* leaves (1.88 units·g^−1^ FW), followed by RL treatment (1.12 units·g^−1^ FW) (Zeng et al., 2023). Our study further confirmed that both BL and RL treatments could promote anthocyanin accumulation in detached L. *ruthenicum* leaves, with BL exhibiting the most pronounced inductive effects (1.34 ± 0.03 units·g^−1^ FW).

Individual and combined treatments of BL and NaCl stress significantly influenced the total anthocyanin content in detached *L. ruthenicum* leaves. The effect of combined application of BL and NaCl was particularly effective, yielding a total anthocyanin content of 3.40 units·g^−1^ FW, which was 2.80-, 1.70-, and 1.40- fold higher than those in the WL treatment, BL treatment, and WL-NaCl treatment, respectively.

Notably, BL and NaCl stress exhibited a significant synergistic effect in promoting anthocyanin accumulation, which was markedly superior to any individual treatment. Those results aligned with existing research indicated that the combined action of BL and osmotic stress synergistically enhanced anthocyanin synthesis in detached L. *ruthenicum* leaves. Therefore, it was hypothesized that this synergistic effect might originate from cross-talk between different signaling pathways, namely BL, as a light signal, activated the expression of light-responsive genes via photoreceptors (e.g., cryptochromes) (Li et al., 2021), while NaCl-induced osmotic stress triggers stress respond through ion toxicity and osmotic signaling pathways (Yu et al., 2023). These pathways collectively acted on key regulatory nodes of the flavonoid biosynthesis pathway, thereby producing an additive or synergistic regulatory effect.

### 4.2 Effects of NaCl and blue light on the flavonoids in detached leaves of L. ruthenicum

UPLC-MS/MS profiles revealed that treatments with both NaCl and BL treatments significantly altered the flavonoid profile in detached L. *ruthenicum* leaves. The results demonstrated that these treatments not only changed the total flavonoid content but also activated specific flavonoid biosynthesis pathways.

Under WL-NaCl treatment, the contents of proanthocyanidins, tannins, biflavonoids, and flavanols increased significantly, with biflavonoid accumulation being especially prominent. The levels of 2,3-dihydroamentoflavone-4″′-methyl ether and 2,3-dihydroamentoflavone-4′-methyl ether were significantly higher than those under BL treatment or the BL-NaCl treatment. Biflavonoids possessed strong hydrophobicity and antioxidant capacity, and their accumulation might represent an adaptive strategy for plants to counteract membrane lipid peroxidation and oxidative damage under NaCl stress (Zhu et al., 2025). This suggested that L. *ruthenicum* leaves might enhance its stress resistance by polymerizing flavonoid units to form structurally more complex and functionally stronger biflavonoids. Furthermore, WL-NaCl treatment promoted the conversion of dihydrokaempferol to dihydromyricetin, followed by subsequent biosynthesis of delphinidin, ultimately catalyzing the production of petunidin and malvidin. Under sucrose osmotic stress, the delphinidin glycoside derivatives in L. *ruthenicum* leaves were differentially upregulated, which was consistent with the results of this study (Wang et al., 2023).

Flavonols are typically acted as UV-shielding agents and photoregulatory substances in plants (Li et al., 2023). Blue light may mimic the effects of some high-energy light (including UV radiation), specifically activate key enzymes in the flavonol synthesis pathway (such as flavonol synthase), thereby significantly promoting flavonols accumulation (Sun et al., 2014). In apples, some health-promoting properties were related to the flavonoids, with flavonols being particularly important (Xie et al., 2020). In New Zealand, apple trees directly exposed to solar ultraviolet light contained more flavonols than those grown under spectral filters that reduced solar ultraviolet transmittance (Henry-Kirk et al., 2018). Quercetin 3-O-glycosides were identified as the main flavonols, and both postharvest UV-B and BL treatment could induce flavonol accumulation in apple peels (Xie et al., 2020). Blue light irradiation also promoted the accumulation of flavonols in three apples (‘Idared’, ‘Fuji’, and ‘Carjevič’), especially the significant variations of quercetin glycosides (Kokalj et al., 2019). BL treatment significantly increased the flavonols content in ginkgo leaves, while RL treatment had the opposite effects (Wang et al., 2022). Blue light significantly increased the flavonoid yield per plant (increased by 0.76 folds) and its antioxidant capacity. Our previous results demonstrated that the *FLS* gene had a significantly higher expression in detached L. *ruthenicum* leaves cultured under BL and BL-sucrose (Zeng et al., 2023). Consistent with these findings, BL treatments in this study markedly promoted the expression of *FLS* genes (**Fig. S4A**), leading to the generation of flavonols, especially the quercetin glycoside derivatives. Additionally, BL treatment promoted the conversion of dihydrokaempferol to dihydroquercetin, subsequently catalyzing the generation of cyanidin, and ultimately leading to significant accumulation of cyanidin-3-O-(6”-O-p-coumaroyl)glucoside, which indicated that this anthocyanin was a light-induced component.

Under the combined treatment of BL and NaCl, the accumulation of flavones, anthocyanins, dihydroflavones, aurones, and chalcones was significantly enhanced. The contents of chalcones and aurones under BL treatment were significantly higher than those under WL-NaCl treatment, indicating that BL signaling was more effective than NaCl stress in activating the expression of *CHS* gene to generate these compounds. The combined treatment further increased the content of these components, suggesting complementary and additive effects of BL and NaCl treatment in regulating flavonoid metabolic flux. In addition, chalcone was the first key intermediate in flavonoid synthesis, and its significant accumulation under BL treatment suggested that the source of the entire biosynthesis pathway was significantly activated. WL-NaCl treatment significantly promoted the conversion of naringenin to eriodictyol, leading to the formation of eriodictyol-7-O-(6“-malonyl) glucoside and eriodictyol-7-O-(6”-acetyl) glucoside, whereas the effects of BL treatment were just the opposite. Next, eriodictyol was catalyzed by flavonoid 3′, 5′-hydroxylase to generate dihydrotricetin, which was further converted by flavanone-3-hydroxylase to generate dihydromyricetin. The differential metabolites induced by BL and NaCl were primarily identified as flavonols and anthocyanins. Under the combined treatments, the contents of differential flavones and flavonols were significantly higher than any single treatment, demonstrating a synergistic promoting effect. Notably, the contents of kaempferol glycoside derivatives under the combined treatment were higher than that of quercetin glycoside derivatives, suggesting that the addition of NaCl might inhibit the conversion of dihydrokaempferol to dihydroquercetin, instead promoting the biosynthesis of kaempferol glycoside derivatives and dihydromyricetin. Furthermore, in NaCl-treated leaves, regardless of being cultured under WL or BL treatment, NaCl significantly promoted the conversion of dihydrokaempferol to dihydromyricetin, while it strongly suppressed the formation of dihydroquercetin from dihydrokaempferol. In addition, our results from sucrose-induced osmotic stress treatment indicated that osmotic stress significantly promoted the expression of the *F3’5′H* gene, leading to the conversion of dihydrokaempferol to dihydromyricetin. In our study, the *F3’5′H* gene (**Fig. S4B and Fig. S4C**) exhibited a higher expression under WL-NaCl treatments, which promoted the dihydrokaempferol to generate dihydromyricetin. Moreover, the content of petunidin-3-O-(6”-O-p-coumaroyl) rutinoside under WL-NaCl treatment was significantly higher than BL treatment, indicating that WL-NaCl treatment promoted dihydromyricetin to produce delphinidin, which was further converted into petunidin and malvidin. The BL-NaCl treatment further promoted the accumulation of those components, revealing a synergistic effect between BL and NaCl in regulating this pathway. In summary, the combined treatment of BL and NaCl significantly promoted the accumulation of multiple flavonoid components in detached L. *ruthenicum* leaves, exhibiting particularly evident synergistic effects on categories such as chalcones, flavones, anthocyanins, and aurones.

**Fig. 6:**
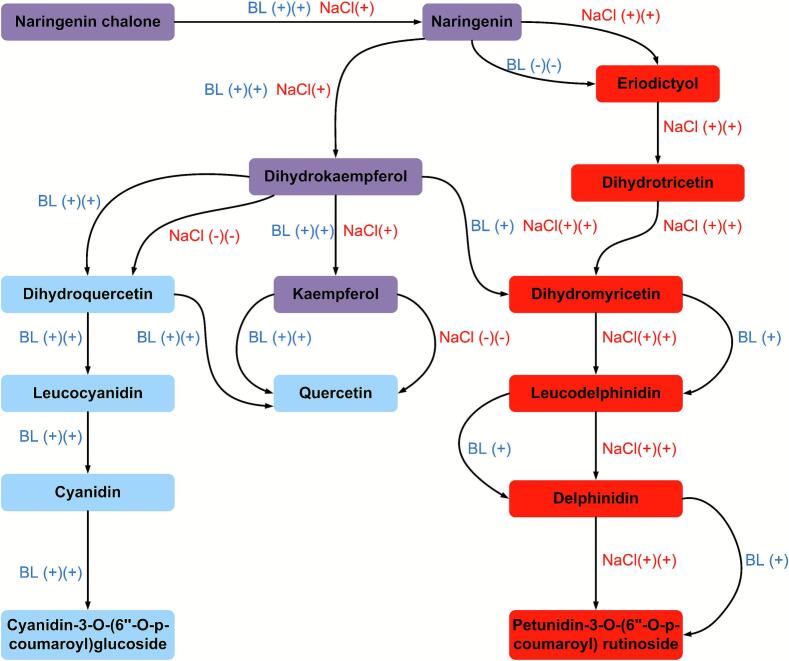
Proposed models exhibiting the regulation of flavonoid biosynthesis in reponse to BL and NaCl treatments.

Based on these findings, we had established comprehensive models that illustrated the independent and synergistic regulatory effects of BL and NaCl treatments on flavonoid biosynthesis in detached L. *ruthenicum* leaves at the metabolomic level (Fig. 6). This advanced the understanding of how light signaling and NaCl stress interact to modulate plant secondary metabolism. The results offered theoretical support and technical pathways for enhancing the biosynthesis and accumulation of high-value flavonoids, especially anthocyanins and flavonols in L. *ruthenicum* leaves through the combined application of blue light irradiation and moderate salt stress. In facility-based cultivation of L. *ruthenicum*, a strategy integrating appropriate NaCl application with supplemental blue LED lighting could be adopted to effectively increase flavonoid content in both leaves and fruits, thereby supporting the objectives of “functional agriculture.” Moreover, using detached leaves or cell culture systems of L. *ruthenicum* cultivated under blue light in a NaCl-supplemented medium displayed promise for developing efficient bioreactor systems to produce high-value flavonoids. Future investigation should focus on elucidating the molecular mechanisms behind these synergistic effects, specifically the expression of key biosynthetic genes and the function of regulatory proteins, to further exploit this system for commercial nutraceutical production.

## 5 Conclusion

In this study, UPLC-MS/MS coupled with chemometrics was employed to systematically investigate the effects of BL and NaCl treatments, both individually and in combination, on flavonoid biosynthesis in detached *L. ruthenicum* leaves. The results clearly demonstrated that both BL and NaCl treatments effectively promoted total anthocyanin accumulation, with a marked synergistic effect observed under BL-NaCl treatment. Furthermore, in-depth metabolic profiling revealed distinct specificity in the regulation of the flavonoid biosynthesis pathway across different treatments. Flavonols and anthocyanins were identified as the predominant classes of differential metabolites in response to BL and NaCl treatments. BL treatment primarily enhanced the flavonols accumulation, whereas NaCl treatment notably induced biflavonoid biosynthesis and promoted naringenin conversion to eriodictyol, thereby enhancing dihydromyricetin biosynthesis. Under the combined BL and NaCl treatment, metabolic flux was more efficiently channeled to the biosynthesis of chalcones, flavones, aurones, and anthocyanins, with a marked stimulation of the conversion of dihydrokaempferol to dihydromyricetin. In summary, this study elucidated the molecular basis of the synergistic regulation of flavonoid biosynthesis induced by BL and NaCl in L. *ruthenicum* at the metabolic level. Our findings provided a solid theoretical foundation and practical guidance for developing efficient strategies that combine light quality regulation with moderate NaCl stress in controlled environments, aiming to enhance the production of high-value bioactive compounds in L. *ruthenicum* to meet the growing demands in functional food and nutritional health products.

## CRediT authorship contribution statement

**Haitao Zeng:** Project administration, Investigation, Funding acquisition, Conceptualization. **Wentao Yang:** Visualization, Resources, Investigation, Conceptualization. **Hao Xu:** Investigation. **Tiantian Chen:** Resources, Investigation, Data curation. **Guodong Wang:** Supervision, Methodology. **Tong Li:** Writing – review & editing, Data curation. **Zhubing Hu:** Software, Methodology. **Tao Zheng:** Writing – original draft, Investigation, Funding acquisition.

## Declaration of competing interest

The authors declare that they have no known competing financial interests or personal relationships that could have appeared to influence the work reported in this paper.

## Data Availability

All the flavonoid profiles under different treatment described in this study were availably obtained on request from the corresponding author.

## Appendix A Supplementary data

Supplementary material 1 Supplementary material 1

Supplementary Table S1 495 flavonoid metabolites detected across the 4 treatment groups. Supplementary Table S1

Supplementary Table S2 259 differential metabolites in the comparison between WL and BL treatments. Supplementary Table S2

Supplementary Table S3 187 differential metabolites in the comparison between WL-NaCl and BL-NaCl treatments. Supplementary Table S3

Supplementary Table S4 191 differential metabolites in the comparison between BL and BL-NaCl treatments. Supplementary Table S4

Supplementary Table S5 242 differential metabolites in the comparison between WL and WL-NaCl treatments. Supplementary Table S5

Supplementary Table S6 260 differential metabolites in the comparison between WL-NaCl and BL treatments. Supplementary Table S6

Supplementary Table S7 19 differential metabolites under combined treatment with BL and NaCl. Supplementary Table S7
