# Supplementary material for: Elucidating the effects of blue light and NaCl on flavonoid biosynthesis in detached Lycium ruthenicum leaves by UPLC-MS/MS and chemometrics
Source: Food Chem X. 2026 Jan 10;33:103507. doi: 10.1016/j.fochx.2026.103507 (PMC12853042; doi:10.1016/j.fochx.2026.103507)
Supplement: Supplementary material 1 [file mmc1.docx]

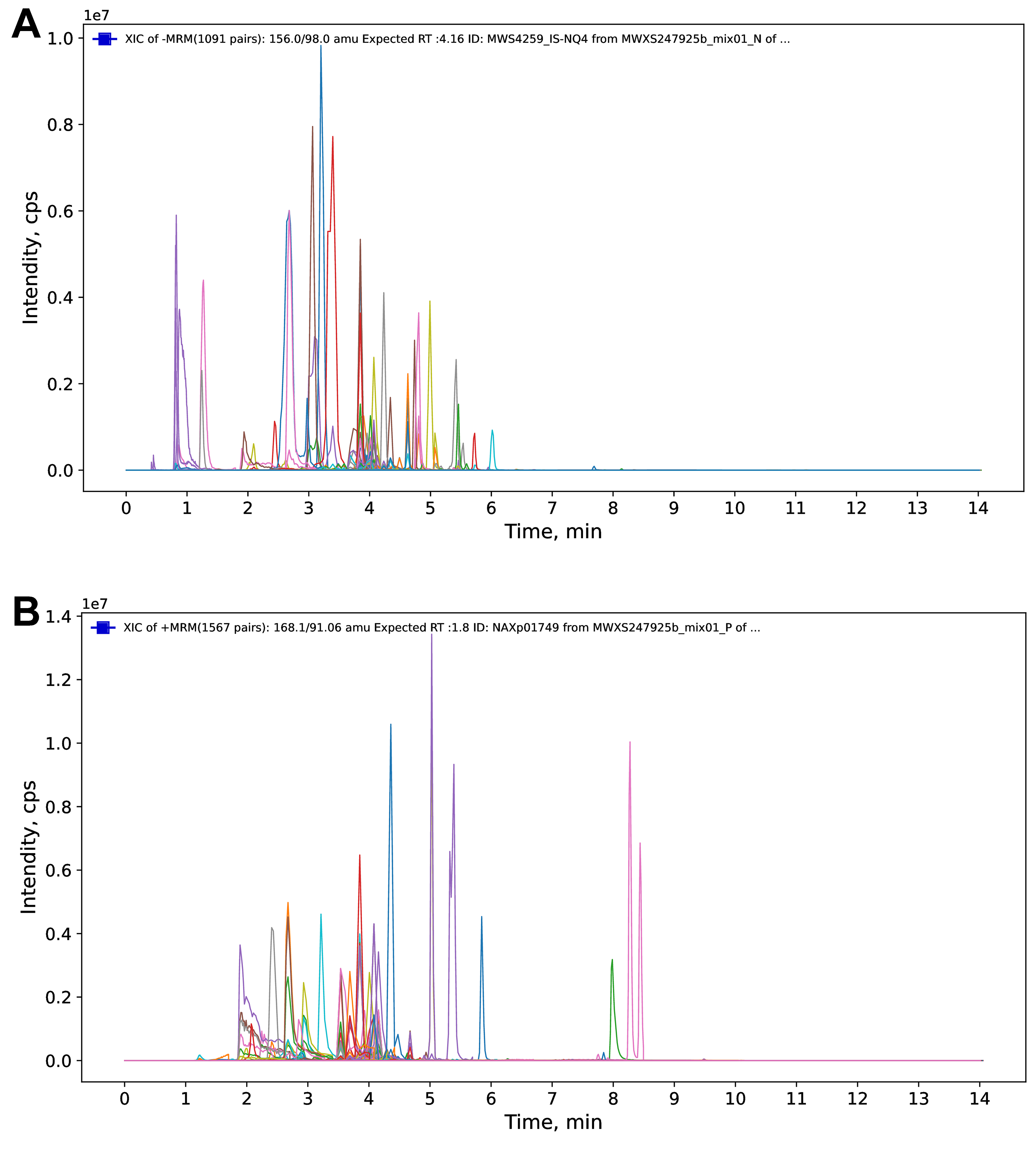


**Fig.S1**. **A**: Mass spectrometry analysis of MRM detection of multimodal map-P. **B**: Mass spectrometry analysis of MRM detection of multimodal map-P.


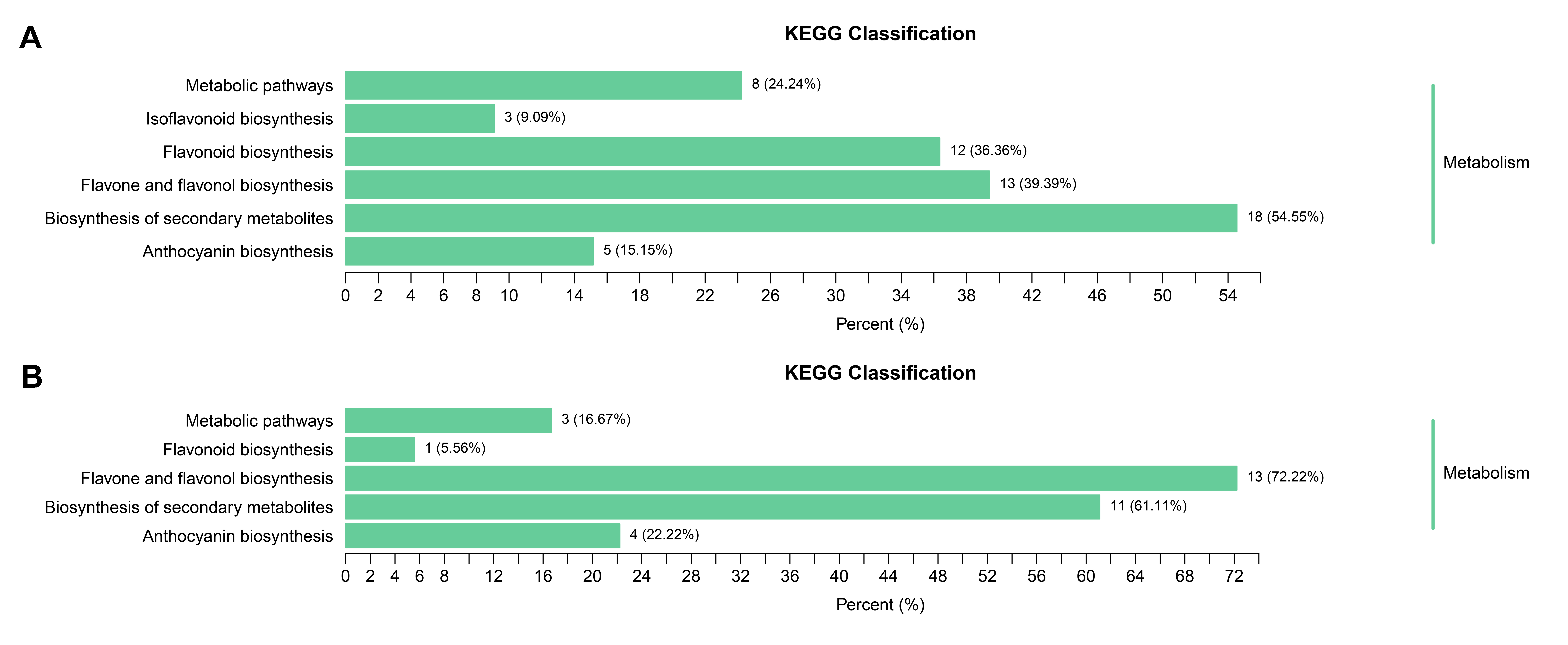


**Fig.S2**. **A**: The KEGG pathway between WL and BL treatments. **B**: The KEGG pathway between WL-NaCl and BL-NaCl treatments.


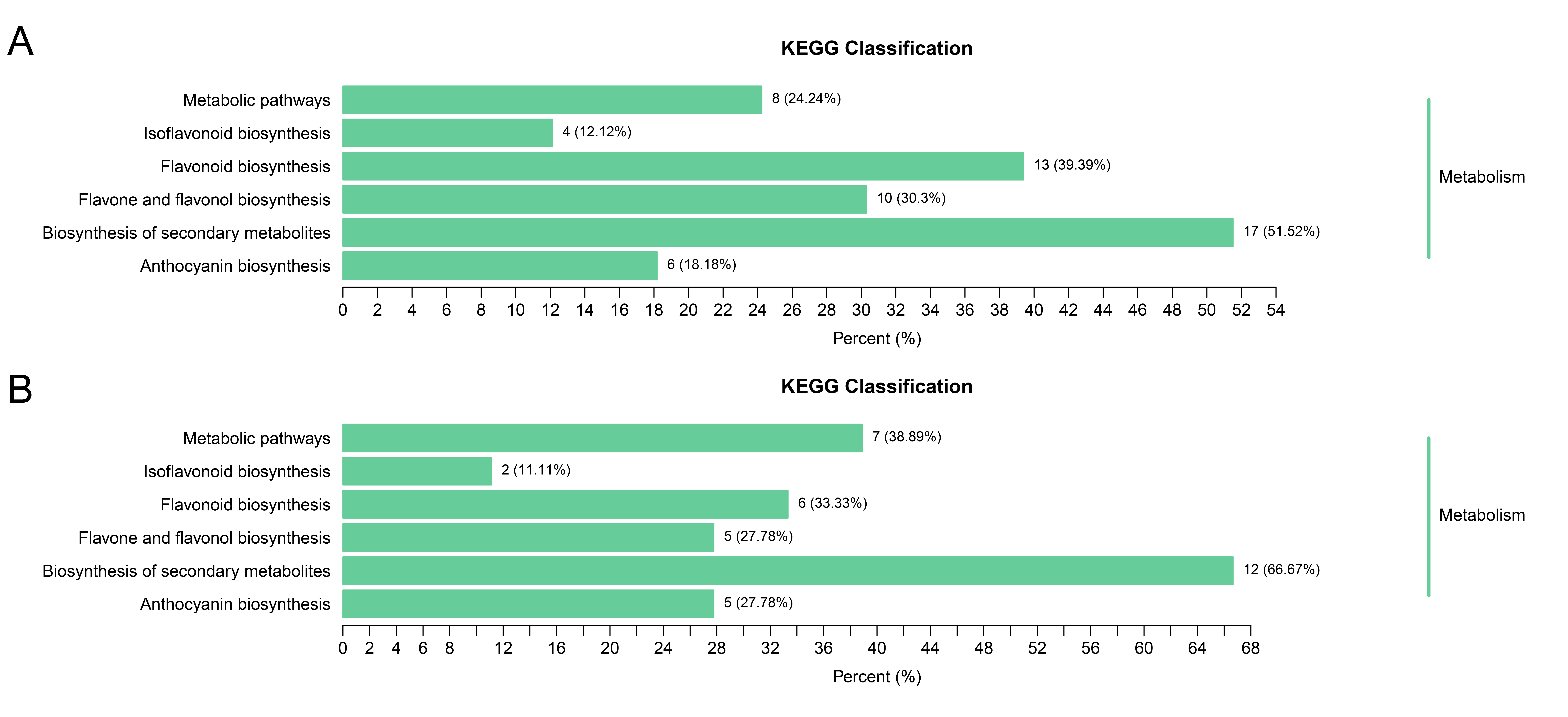


**Fig.S3**. **A**: The KEGG pathway between WL-NaCl and WL treatments. **B**: The KEGG pathway between BL -NaCl and BL treatments.


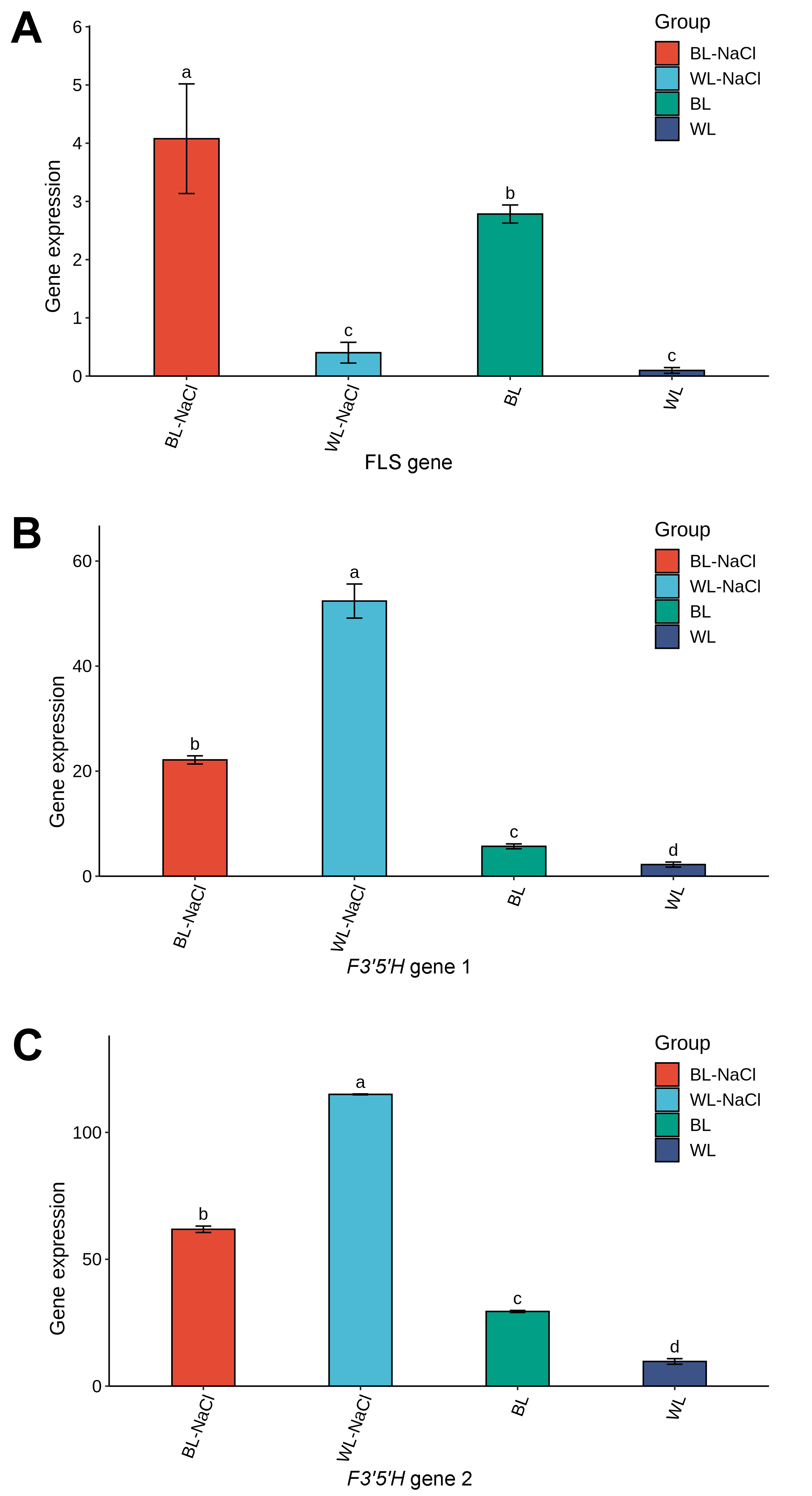


**Fig.S4**. The *FLS* and *F3'5'H* gene expression under 4 treatments. **A**: The expression of *FLS* gene. **B**: The expression of *F3'5'H* gene 1. **C**: The expression of *F3'5'H* gene 2.
